# The Genome Sequence of Alpine *Megacarpaea delavayi* Identifies Species-Specific Whole-Genome Duplication

**DOI:** 10.3389/fgene.2020.00812

**Published:** 2020-08-03

**Authors:** Qiao Yang, Hao Bi, Wenjie Yang, Ting Li, Jiebei Jiang, Lei Zhang, Jianquan Liu, Quanjun Hu

**Affiliations:** ^1^Key Laboratory of Bio-Resource and Eco-Environment of Ministry of Education, State Key Laboratory of Hydraulics and Mountain River Engineering, College of Life Sciences, Sichuan University, Chengdu, China; ^2^State Key Laboratory of Grassland Agro-Ecosystem, Institute of Innovation Ecology, Lanzhou University, Lanzhou, China

**Keywords:** *Megacarpaea delavayi*, genome sequence, alpine adaptation, whole-genome duplication, Brassicaceae

## Abstract

*Megacarpaea delavayi* (Brassicaceae), a plant found the high mountains of southwest China at high altitudes (3000–4800 m), is used as a vegetable or medicine. Here, we report a draft genome for this species. The assembly genome of *M. delavayi* is 883 Mb, and 61.59% of the genome is composed of repeat sequences. Annotation of the genome identified a total of 41,114 protein-coding genes. We found that *M. delavayi* experienced an independent whole-genome duplication (WGD), paralleling those independent WGDs in *Iberis*, *Biscutella*, and *Anastatica* in the early Miocene. Phylogenetic analyses based on the single-copy genes confirmed the position of the genus *Megacarpaea* within the expanded lineage II of the family and resolved its basal divergence to a subclade consisting of *Anastatica, Iberis*, and *Biscutella.* Species-specific and fast-evolving genes in *M. delavayi* are mainly involved in “DNA repair” and “response to UV-B radiation.” These genetic changes may together help this species survive in high-altitude environments. The reference genome reported here provides a valuable resource for studying adaptation of this and other alpine plants to the high-altitude habitats.

## Introduction

Polyploidy (whole-genome duplication, WGD), which occurs frequently through evolutionary histories of plants (Wu et al., 2020), contributes greatly to both species diversification and colonization of the new niches (Soltis and Soltis, 2016). Numerous independent WGDs were identified for angiosperm families, such as Asteraceae (Huang et al., 2016), Poaceae (McKain et al., 2016), and Brassicaceae (Edger et al., 2015). The Brassicaceae-specific WGD was named At-α WGDs (Bowers et al., 2003), which occurred 23–43 million years ago (Mya) (Mandáková et al., 2010). In addition, more independent WGDs were revealed to be specific to lineages or species within the family (Mandáková et al., 2017). It is increasingly clear that subsequent lineage-specific or species-specific WGD events laid the foundation for species diversification, environmental adaptability, and stress tolerance of the Brassicaceae species (Kagale et al., 2014).

In this study, we aimed to examine whether WGD occurred in one alpine herb, *Megacarpaea delavayi* (2*n* = 18) of Brassicaceae through sequencing its draft genome. This species grows on swampy meadows, steep grassy slopes, and open thickets of the high mountains (Hengduan Mountains) in southwest China at elevations of 3000–4800 m (Cheo et al., 2001). It has been collected as a wild vegetable and medicine for years by the local inhabitants in the high-altitude regions (Zhong et al., 2015). The dried plants of *M. delavayi* are used to treat dysentery, lung cough, and disordered indigestion by Bai and Tibetan people (Lei et al., 2009; Shen et al., 2009). Other species of the genus are distributed in the high-elevation regions from the Hengduan Mountains to Himalaya and central Asia (Cheo et al., 2001). However, phylogenetic relationships of the genus *Megacarpae* in the family Brassicaceae remain unclear although both recent studies suggested its likely position in the expanded lineage II of the family Brassicaceae (Guo et al., 2017; Nikolov et al., 2019). Maximum likelihood analyses based on targeted enrichment sequence data suggested the close relationship between *Megacarpaea*, *Iberis*, *Cochlearia* and others although this received little support according to coalescent analyses of these data (Nikolov et al., 2019). In addition, in these analyses, it remained unsolved whether *Biscutella* should be placed in this clade (Nikolov et al., 2019) although another study based on genome-scale single-copy genes suggested the well-supported close relationship between *Biscutella* and *Iberis* (Kiefer et al., 2019). Independent WGDs, which might have led to incorrect gene orthology alignments (Walker et al., 2017), seem to account for these conflicting phylogenies (Nikolov et al., 2019).

Here, we report the assembly and comparative genomic analysis of the *M. delavayi* genome. We revealed that one independent WGD occurred in this species in the early Miocene, paralleling those WGDs in other genera. We determined the phylogenetic relationship of the genus *Megacarpaea* based on genome-scale single-copy genes. We further found that numerous species-specific and fast-evolving genes existed in this species, which may be beneficial for its survival in the alpine habitat.

## Materials and Methods

### Plant Materials and Genomic DNA Extraction

**FIGURE 1:**
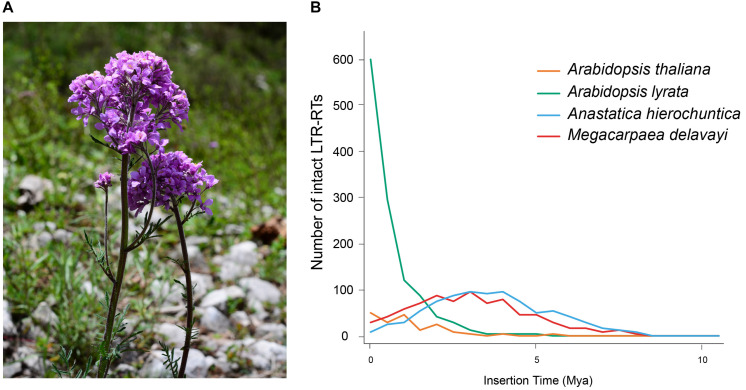
**(A)** *M. delavayi* in flowering stage of growth. Photo taken by Lei Zhang in Pingwu County (3,077 m a.s.l.), Sichuan Province, China. **(B)** Insertion time distribution of intact LTR-RTs of *M. delavayi* and other Brassicaceae species.

One wild *M. delavayi* (2*n* = 18) individual (Figure 1A) was collected from Cangshan Mountain (3181 m, N25.659, E100.117) in Yunnan Province, China. Fresh and healthy leaves were immediately frozen at −80°C for DNA extraction. High-quality genomic DNA from leaf tissue was extracted with the CTAB method (Liu et al., 2009). We used 1% agarose gel electrophoresis to check the quality of the high-molecular-weight DNA. High-quality genomic DNA with an effective concentration of more than 2 nM was used to construct the library.

### Genome Sequencing and Assembly

We constructed Illumina paired-end libraries with small (230, 500, and 800 bp) and large (2, 5, 10, and 20 kb) insert sizes and read lengths of 150 bp (Supplementary Table S1). We sequenced them using the Illumina HiSeq 2500 platform (Illumina, San Diego, CA, United States) at Novogene (Tianjin, China) following the manufacturer protocols. Short reads were first subjected to quality filtering with Trimmomatic v0.36 (Bolger et al., 2014), error correction with BFC v1.8 (Li, 2015), and mate-pair data deduplication with FastUniq v1.1 (Xu et al., 2012). The genome was initially assembled into scaffolds with Platanus v1.2.4 (Kajitani et al., 2014) with the first round of gap closing. An additional gap closing procedure was performed with GapCloser v1.12 (Luo et al., 2012). Finally, we evaluated genome assemblies for completeness using BUSCO v3.0.2 (Waterhouse et al., 2018) with “embryophyta_odb9” (Supplementary Table S2).

### Genome Size Estimation

The sequencing reads were used to estimate genome size of *M. delavayi* based on k-mer frequencies. We used quality-filtered Illumina short reads and 17-mer frequency distribution to estimate the genome size with Jellyfish v2.2.9 (Marçais and Kingsford, 2011). The highest peak value of k-mer distribution was used to estimate the sequencing depth. We plotted the distribution of k-mer depth against the frequency with the main peak occurring at a depth of 19 (Supplementary Figure S1). A Perl script^1^ was used to calculate the size of the *M. delavayi* genome.

### Repetitive Identification

We used a combination of homology-based and *de novo* search methods to identify repeat sequences in the *M. delavayi* genome. In terms of homology-based prediction, RepeatMasker v4.0.7 (Tarailo-Graovac and Chen, 2009) was used to find repeat elements at the DNA level with the Repbase library. *De novo* repeat annotation of the *M. delavayi* genome was performed with RepeatModeler v1.0.11 (Smit and Hubley, 2008).

We identified intact long-terminal repeat retrotransposons (LTR-RTs) by searching the *M. delavayi* genome with LTRharvest v1.5.10 (Ellinghaus et al., 2008; -motif tgca -motifmis 1) and LTR_Finder v1.06 (Xu and Wang, 2007; -D 20000 -d 1000 -L 5000 -l 100). Then, LTR_retriever v1.9 (Ou and Jiang, 2018) was used to integrate the results of LTR_Finder and LTRharvest (Supplementary Table S3). Using a substitution rate (*r*) of 7 × 10^–9^ substitutions per site per year (Ossowski et al., 2010), we calculated the insertion time (*T*) for each LTR retrotransposon as *T* = *K*/(2*r*), where *K* is genetic distance and *r* is the rate of nucleotide substitution per site per year (*r* = 7 × 10^–9^).

### Gene Annotation

A combination of *de novo*, homology-based, and transcript-based approaches were used to predict protein-coding genes in the *M. delavayi* genome. Before the transcriptome could be aligned, RNA-Seq reads needed to be assembled into transcripts. We first filtered RNA-Seq reads with potential low-quality regions using Trimmomatic v0.36 (Bolger et al., 2014). After quality control was performed, all clean reads were assembled into *de novo* transcripts with Trinity v2.8.4 (Haas et al., 2013). Then, we used PASA v2.1.0 (Haas et al., 2003) to obtain information on the gene structure annotation by aligning the assembled transcripts with the genomes. Protein sequences of *Aethionema arabicum*, *Arabidopsis lyrata*, *Arabidopsis thaliana*, *Brassica rapa*, and *Capsella rubella*, were obtained for homology-based gene annotation. GlimmerHMM v3.0.4 (Majoros et al., 2004) was used to predict the gene structure in each protein-coding region. We performed *de novo* prediction with AUGUSTUS v3.2.3 (Stanke et al., 2006) to annotate protein-coding genes. The gene model parameters were trained from *A. thaliana* and our transcriptome data set. The above three gene prediction results were merged with EVidenceModeler v1.1.1 (Haas et al., 2008) to form a comprehensive and non-redundant reference gene list. Weights of evidence for gene models were defined as follows: *de novo* prediction weight (Augustus) = 1, homology-based prediction weight (GlimmerHMM) = 5, transcript-based prediction weight (PASA) = 10. The EVM merged result was updated with an additional round of PASA annotation to add UTRs and provide information on alternative splicing variants to gene models.

To obtain functional annotation of protein-coding genes, we used Blast2GO v2.5 (Conesa and Götz, 2008) for gene ontology (GO) annotation based on the NCBI-NR database. The predicted genes were mapped to KEGG pathways using KAAS (Moriya et al., 2007) to obtain the KEGG annotation. For Swiss-Prot annotations, we employed BLAST + v2.2.31 (Camacho et al., 2009; Blastp with the E-value cutoff 1 × 10^−5^) to align proteins to the Swiss-Prot databases. InterProScan v5.31-70 was used to determine the domains/motifs (Jones et al., 2014; Supplementary Table S4).

### Gene Family Identification

We downloaded the protein-coding genes of *Arabis alpina*, *Boechera stricta*, *Crucihimalaya himalaica*, *Eutrema heterophyllum*, and *Lepidium meyenii* together with *Megacarpaea delavayi* to identify orthologous groups (Supplementary Table S5). To remove redundancy caused by alternative splicing variations, we retained only the gene models at each gene locus that encoded the longest protein sequence, and putative fragmented genes that encoded protein sequences shorter than 50 aa and stop codon ratios greater than 20% were filtered out. Then, we used Diamond v0.9.22 (Buchfink et al., 2014; E-value cutoff 1 × 10^−5^) to compare all filtered protein sequences and used OrthoMCL v2.0.9 (Li et al., 2003) to cluster genes into orthologous groups. Genes that could not be clustered into any gene family and for which only one species existed were considered species specific. Finally, we summarized the gene family cluster results for six species in Venn diagram format.

### Phylogenetic Analyses

We downed the reported genomes of all related species from two main lineages of Brassiaceae, including *Aethionema arabicum*, *Anastatica hierochuntica*, *Arabidopsis lyrate*, *Arabidopsis thaliana*, *Biscutella auriculate, Biscutella laevigata, Brassica rapa*, *Boechera stricta, Crucihimalaya himalaica, Eutrema heterophyllum, Iberis amara, Kernera saxatilis, Lepidium meyenii, Macropodium nivale*, and *Noccaea caerulescens* (Supplementary Table S5). We used the single-copy orthologous genes identified in the gene family cluster analyses from these species and *M. delavayi* to construct phylogenetic tree. Multiple sequence alignments were performed for the protein sequence of each single-copy orthologous gene with MAFFT v7.313 (Katoh and Standley, 2013). Then, the alignments were concatenated to generate a super alignment matrix, which was used to generate a maximum likelihood tree with the PROTGAMMAILGX model in RAxML v8.2.11 (Stamatakis, 2014).

The divergence time between these species was estimated with the MCMCtree program in PAML v4.9 (Yang, 2007). The F84 model (model = 4) and independent rates molecular clock (clock = 2) were used for calculations in MCMCtree. The MCMC process was run for 1,500,000 iterations, with a sample frequency of 150, after a burn-in of 500,000 iterations. We ran the program twice for each data type to confirm that the results were convergent between runs. We looked up three calibration points in the TimeTree database (Kumar et al., 2017) to estimate the Brassicaceae divergence time: divergence time for *Ae. arabicum* and other Brassicaceae plants was 32–43 Mya, divergence time for Lineages II+ expanded lineage II and lineage I was 23.4–33.5 Mya, divergence time for *L. meyenii* and other lineage I plants was 11.9–20.6 Mya. The phylogenetic analyses for Brassicaceae was visualized with FigTree v1.4.3^2^.

### Gene Family Expansion and Contraction

CAFÉ (De Bie et al., 2006) is a tool for analyzing evolutionary changes of the gene families. This software uses the stochastic birth and death process to model gene gain and loss over a phylogeny. Based on the results of phylogeny and divergence time, we applied CAFE v4.2 to identify gene families that had undergone expansion and contraction on the phylogeny tree (*p*-value cutoff 0.05). For each significantly expanded and contracted gene family in *M. delavayi*, we inferred functional information based on its functional annotations.

### WGD and Positively Selected Genes

We used MCScanX (Wang et al., 2012) to detect syntenic blocks (regions with at least five collinear genes) and duplication levels (depth) for four species: *A. hierochuntica*, *A. thaliana*, *B. rapa*, and *M. delavayi*. To recover the WGD event, we calculated synonymous substitution rates (Ks) for syntenic genes using codeml in PAML. To further examine whether the recent WGDs were shared by *M. delavayi* and the closely related species, we extracted homologous gene groups to construct phylogenetic trees of the orthologous genes. We used phylogenetic relationships of the homologous genes identified the likely WGD nodes.

To identify fast-evolving genes, we used MCScanX to search for syntenic blocks. Similar to previous research (Wu et al., 2019), we calculated non-synonymous substitution (Ka) and synonymous substitution (Ks) for the collinear orthologous gene pairs using the Perl script “add_ka_and_ks_to_collinearity.pl” in MCScanX. The ratio of Ka to Ks is a commonly used indicator of selective pressure acting on protein-coding genes with a ratio >1 representing positive selection.

## Results

### Genome Size Estimation

The distribution of short subsequence (k-mer) frequency, also known as the k-mer spectrum, is widely used to estimate genome size (Li et al., 2013; Zhang et al., 2015). A k-mer depth distribution was obtained from Jellyfish (Marçais and Kingsford, 2011) analyses, and the peak depth was clearly visible from the distribution data (Supplementary Figure S1). The genome size of *M. delavayi* calculated by the aforementioned Perl script was estimated to be approximately 899 Mb (Table 1).

**TABLE 1:** Statistics for the *M. delavayi* genome assembly and annotation.

Feature	Value
Estimated size (Mb)	899
Assembly size (Mb)	883.81
Number of contigs	763,815
Maximum contig length (kb)	794.13
Contig N50 (kb)	65.48
Number of contigs at least N50	3,402
GC content (%)	31.64
Repeat content (%)	61.59
Number of protein-coding genes	41,114
Average gene length (bp)	1,852.54
Average exon length (bp)	266.06
Average number of exons per gene	6.57

### Genome Assembly and Annotation

To sequence the whole genome of *M. delavayi*, we generated 133 Gb of paired-end and mate-pair clean reads (150 × assembled sequence coverage) with different insert sizes using an Illumina HiSeq 2500 platform (Supplementary Table S1). The final assembly genome of *M. delavayi* (883.81 Mb) consisted of 763,815 contigs (contig N50, 65.48 kb; longest contig, 794.13 kb; Table 1). Completeness of the genome regions was further assessed with BUSCO. Of a core set of 1440 single-copy ortholog genes of the Embryophyta lineage, 94.5% were complete in the genome, which contained 85.6% of the plant single-copy orthologs and 8.9% of the plant duplicate orthologs (Supplementary Table S2), which suggests that the genome of *M. delavayi* was well-assembled with high completeness and accuracy.

*De novo*, homology-based, and transcript-based approaches were combined to annotate protein-coding sequences (Chen et al., 2020; Kang et al., 2020; Zhang et al., 2020). In total, 41,114 genes were predicted with an average gene length and number of exons of 1853 bp and 6.57, respectively (Table 1). Moreover, we annotated functions of the predicted genes with Swiss-Prot, InterProScan, GO, and KEGG databases. The results showed that 85.78% of all the protein-coding genes were successfully annotated by at least one database (Supplementary Table S4).

### Repetitive Elements Analysis

Through combination of *de novo* searches and homology-based methods, we identified nearly 536 Mb repetitive elements, representing 61.59% of the *M. delavayi* genome (Table 2). Retrotransposons (LTR, SINEs, and LINEs) were the most abundant, accounting for 44.06% of the genome. LTR-RTs represented 39.25% of the genome, and Ty3/Gypsy (24.71%) made up major elements of LTR-RTs (Table 2).

**TABLE 2:** Repeat content (subtypes) of *M. delavayi* genome.

Repeat type	Repeat size (bp)	Percentage of genome (%)
DNA transposons	103,382,966	11.70
LTR retroelements:	346,878,665	39.25
Ty1/Copia	20,388,180	2.31
Ty3/Gypsy	218,392,577	24.71
SINE retroelements	1,084,358	0.12
LINE retroelements	41,410,409	4.69
TRF	27,943,342	3.16
Simple repeats	6,422,772	0.73
Low complexity	718,144	0.08
Unclassified	9,975,172	1.13
Unknown	6,485,326	0.73
Total	536,335,637	61.59

A high proportion of repetitive elements in the *M. delavayi* genome were LTR-RTs, and the proliferation of retrotransposons might have been responsible for genome expansion (Zhang et al., 2019). To estimate insertion time of the LTR-RTs, we identified complete LTR-RTs in four Brassicaceae species (*A. hierochuntica*, *A. thaliana*, *A. lyrate*, and *M. delavayi*). We identified 814 complete LTR-RTs in the *M. delavayi* genome and 176 in *A. thaliana* and 1227 in *A. lyrate* (Supplementary Table S3). *A. thaliana* had more microdeletions in transposons than *M. delavayi* (Hu et al., 2011), and *A. lyrata* had a comparatively higher proportion of recent insertions (Slotte et al., 2013), consistent with our study. The insertion time distribution showed that *M. delavayi* LTR-RTs expanded within the past 5 million years (based on *r* of 7 × 10^–9^ substitutions per site per year; Figure 1B). In general, recent expansion of repeat sequences may have played a key role in increasing the genome size of *M. delavayi*.

### Phylogenetic Analyses

**FIGURE 2:**
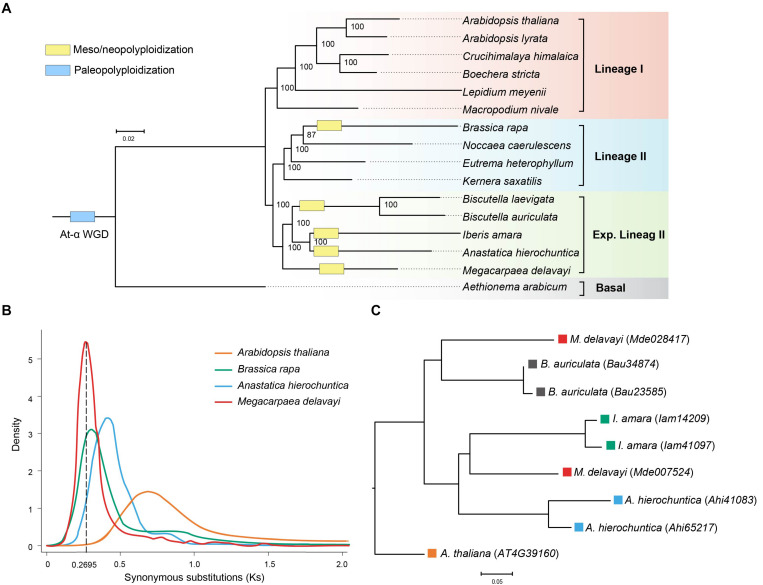
**(A)** Phylogenetic relationship of *M. delavayi* and other Brassicaceae species. Numbers on nodes represent bootstrap values. **(B)** Synonymous substitutions (Ks) estimated from *M. delavayi*-specific WGD. **(C)** A phylogenetic tree of the homologous genes from the expanded lineage II.

Sixteen species were selected to identify orthologous groups, and they were clustered into 51,589 orthologous groups. A total of 361 single-copy gene families were identified and used to construct the maximum likelihood phylogenetic tree. Three major lineages were identified: lineages I, and traditionally recognized lineages II and those added to the expanded lineage II. Phylogenetic analysis confirmed the phylogenetic position of *M. delavayi* in the expanded lineage II of Brassicaceae (Figure 2A), consistent with previous published research (Guo et al., 2017; Kiefer et al., 2019; Nikolov et al., 2019). Within this lineage, *M. delavayi* diverged early as one subclade, and another comprised *Anastatica*, *Iberis* and *Biscutella* as suggested before based on the genome-scale single-copy genes. The close relationships between the latter three genera agree with phylogenetic analyses similarly based on the genome-scale, single-copy genes (Kiefer et al., 2019). Based the fossil-calibrated phylogeny, *M. delavayi* diverged from other three genera of the expanded lineage II around 20.53 Mya (Supplementary Figure S2).

### Whole-Genome Duplication

The distribution of Ks was analyzed to uncover and assess the frequency of WGD events in the Brassicaceae (Kagale et al., 2014). Using syntenic orthologs within each genome to construct the distribution of Ks, we found *M. delavayi* had undergone a further more species-specific WGD (Figure 2B) after the well-known ancient At-α (23–47 Mya) paleopolyploid WGD. Although the Brassicaceae diverged from other closely related eudicots at the beginning of the Cenozoic era, the rapid species diversification of the family occurred only within the Miocene (<23 Mya; Kagale et al., 2014). Importantly, the lineage- or species-specific polyploid or WGD events seemed to have promoted species diversification during this recent stage (Kagale et al., 2014; Kiefer et al., 2019). Four genera, *Brassica*, *Anastatica*, *Iberis*, and *Biscutella*, of the expanded lineage II were suggested to experience independent WGDs (Wang et al., 2011; Kiefer et al., 2019). We examined Ks distributions of these species. We confirmed these WGDs and found that they occurred between 16 and 23 Mya almost at the same stage after their divergences (Figure 2B). These WGDs seem to occur independently based on the Ks distributions despite the slight differences between them. It remains interesting to further examine whether *Megacarpaea* shared a WGD with the closely related *Anastatica*, *Iberis*, and *Biscutella* based the homologous genes. After the WGD, the duplicated gene may have been randomly lost in the derived lineages, and therefore, it is difficult to identify all paralogous genes between different lineages. We, therefore, used *Arabidopsis* without further WGD as an out-group. We extracted a set of homologous gene groups: *Arabidopsis* (1): *Megacarpaea* (2): *Anastatica* (2): *Iberis* (2): *Biscutella* (2). We recovered 24 groups of homologous genes, but phylogenetic analyses suggested that only one group could be used to construct a gene tree with most subclades statistically supported (Figure 2C). On this tree, two genes from *Megacarpaea* did not cluster together, and their clustering with other subclades failed to receive statistical support. Because two genes from each of the other three genera comprised a monophyletic clade, respectively, it is highly likely that WGDs occurred in these genera independently.

### Gene Family Expansion and Contraction

Gene loss and gain are the primary reasons for functional changes (Xing et al., 2019). To better understand the relationships between the gene families of *M. delavayi* and other crucifer, we performed a systematic comparison of genes among different species. Phylogenetic analyses indicated that *M. delavayi* was phylogenetically categorized into expanded lineage II. Further comparisons of these species revealed 2673 expanded and 3600 contracted gene families in the *M. delavayi* genome. Significant expansion or contraction in the size of particular gene families is often associated with the adaptive divergence of related species (Zhang et al., 2016). Also, a total of 41 gene families showed significant expansion (*P* < 0.05), and 37 gene families showed significant contraction (*P* < 0.05) in *M. delavayi*. The significantly expanded gene families contained 312 genes, which are mainly involved in “response to light,” “response to salt stress,” “response to water deprivation,” and “calcium-mediated signaling” (Supplementary Table S6). This also agrees with the previous predication that some species from the expanded lineage II of Brassicaceae are salt-tolerant (Monihan et al., 2020).

**FIGURE 3:**
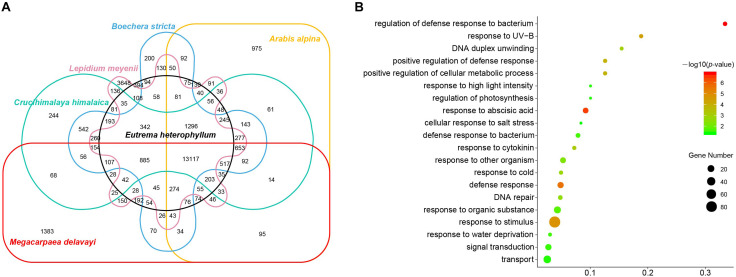
**(A)** Venn diagram showing shared and unique gene families among six species of Brassicaceae. **(B)** Gene ontology (GO) enrichment of *M. delavayi*–specific genes.

We further examined the shared and species-specific gene families between *M. delavayi* and other alpine crucifers with genomes available: *A. alpine* (Willing et al., 2015), *B. stricta*, *C. himalaica* (Zhang et al., 2019), *E. heterophyllum* (Guo et al., 2018), and *L. meyenii* (Zhang et al., 2016). These alpine crucifers were found to develop more species-specific genes to adapt to alpine habitats. We next examined whether *M. delavayi* developed more such genes in addition to those shared with other alpine crucifers. We identified a total of 28,835 homologous gene families, 13,117 of which were shared by six alpine crucifers. We identified 1383 gene families specific to the *M. delavayi* genome (Figure 3A). GO enrichments of these genes in species-specific gene families revealed that they were mainly involved in “response to UV-B,” “response to cold,” “DNA repair,” and “cellular response to salt stress” (Figure 3B and Supplementary Table S7).

### Fast-Evolving Genes in *M. delavayi*

In plants, tolerance to UV-B radiation and cold are critical for surviving at high altitudes (Zhang et al., 2016). Increased Ka relative to Ks in certain genes may explain the adaptive evolution of organisms at the molecular level (Qiu et al., 2012). We identified 1203 syntenic gene blocks containing 20,442 collinear gene pairs in the *M. delavayi* and *A. thaliana* genomes. A total of 327 genes under positive selection had a Ka/Ks ratio greater than 1.0. The Swiss-Port functional classification revealed that the fast-evolving genes with putative functions were related to “DNA repair,” “response to UV-B radiation,” “defense response,” and “response to cold” (Supplementary Table S8). In particular, we found that *DRT101* (*Mde002296.11*) related to the DNA repair from UV damage evolved quickly in the *M. delavayi* genome (Supplementary Table S8). In the maca genome, genes related to DNA repair (*DRT102*) have also been found to evolve rapidly (Zhang et al., 2016). Both *DRT101* and *DRT102* belong to the DNA-damage-repair/toleration (*DRT*) genes (Supplementary Table S9), and they may encode UV-specific excision repair activities (Pang et al., 1993; Hays and Pang, 1994). The accelerated evolution of DRT and other genes may help *M. delavayi* adapt to the high-altitude environment.

## Discussion

In this study, we performed *de novo* assembly of the *M. delavayi* genome based on an Illumina HiSeq 2500 platform. *M. delavayi* is the first sequenced species of the genus *Megacarpaea*. This reference genome provides a basis for further studying speciation based on genomic data for the genus and comparative genomics studies in the family Brassicaceae. The genome of *M. delavayi* was estimated at 899 Mb, and the final assembly genome was 883.81 Mb, representing about 98% of the estimated genome size (Table 1). WGD and expansion of repetitive elements in the *M. delavayi* genome might have led to its larger size than other species (Figure 1B and Supplementary Table S3). Based on the genome-scale single copy genes, our phylogenetic analysis clearly shows that *Megacarpaea* was placed in the expanded lineage II of Brassiaceae, and it was closely related to *Anastatica*, *Iberis*, and *Biscutella*. This finding resolved the ambiguous phylogenetic position of the genus *Megacarpaea* in the previous study (Nikolov et al., 2019) because of the difficulties in gene orthologous alignments (Walker et al., 2017).

Our genomic analyses suggested one species-specific WGD event in the *M. delavayi* genome. This WGD paralleled independently to those that occurred in the closely related species, *Anastatica*, *Biscutella*, and *Iberis.* These WGDs were estimated to occur within the Miocene shortly after the radiative divergences of the sampled genera. Such repeated WGDs accompanying lineage divergences together drove species diversification of the family (Kagale et al., 2014). In addition, WGDs should have also played an important role for crucifers to colonize the arid habitats because of the obvious advantages of the polyploids under selective pressure (Dong et al., 2019).

Compared with the published alpine crucifers with available genomes (Guo et al., 2018; Zeng et al., 2019; Zhang et al., 2019), we also found that *M. delavayi* retained numerous species-specific genes, which are involved in “response to UV-B,” “response to cold,” “DNA repair,” and “cellular response to salt stress” (Figure 3B and Supplementary Table S7). These genes derived from WGD or other ways may also play an important role for *M. delavayi* to adapt to a cold and UV-B stressed habitat at high altitude (Cheviron and Brumfield, 2012). In addition, fast-evolving genes in *M. delavayi* were also found to be involved in “DNA repair” and abiotic stresses (Supplementary Table S8). All these findings suggest that *M. delavayi* had developed obvious genomic changes to adapt to alpine habitats. The reference genome presented here provides an important resource for further studying molecular adaptation of this and other alpine plants to the highlands.

## Data Availability Statement

The genomic sequence data of *Megacarpaea delavayi* in this study have been deposited in the NCBI under BioProject PRJNA630110. The assembled genome and genome annotation information have been deposited in the National Genomics Data Center (https://bigd.big.ac.cn/?lang=zh) under BioProject PRJCA002887.

## Author Contributions

QH and JL designed the research. LZ and HB collected the materials and performed the genome sequencing and assembly. QY, WY, HB, TL, JJ, and LZ performed the genome annotation and evolution analyses. QY, QH, and JL wrote the manuscript. All authors contributed to the article and approved the submitted version.

## Conflict of Interest

The authors declare that the research was conducted in the absence of any commercial or financial relationships that could be construed as a potential conflict of interest.

## Footnotes

1 https://github.com/josephryan/estimate_genome_size.pl

2 http://tree.bio.ed.ac.uk/

## Supplementary Material

FIGURE S1 17-k-mer frequency distribution of sequencing reads from *M. delavayi*. The size of the *M. delavayi* genome was estimated at 899 Mb.

FIGURE S2 Phylogenetic tree with divergence time and expanded/contracted gene family for *M. delavayi* and other Brassicaceae species. The numbers above the branches are the predicted divergence times.

TABLE S1 Sequencing statistics of the *M. delavayi* genome.

TABLE S2 Evaluation *M. delavayi* genome assembly with BUSCO.

TABLE S3 Numbers of annotated protein-coding genes in *M. delavayi* genome.

TABLE S4 Intact LTR-RTs in *M. delavayi* and other Brassicaceae species.

TABLE S5 Protein data sets used for comparative genomic analyses.

TABLE S6 Function of significantly expanded genes in *M. delavayi* genome.

TABLE S7 GO enrichment of *M. delavayi* specific genes.

TABLE S8 Function of fast-evolving genes in *M. delavayi* genome.

TABLE S9 DRT genes copy number in *M. delavayi* and *L. meyenii* genomes (identity > 80%).
