# Supplementary figures and images for: The Genome Sequence of Alpine Megacarpaea delavayi Identifies Species-Specific Whole-Genome Duplication
Source: Front Genet. 2020 Aug 3;11:812. doi: 10.3389/fgene.2020.00812 (PMC7416671; doi:10.3389/fgene.2020.00812)

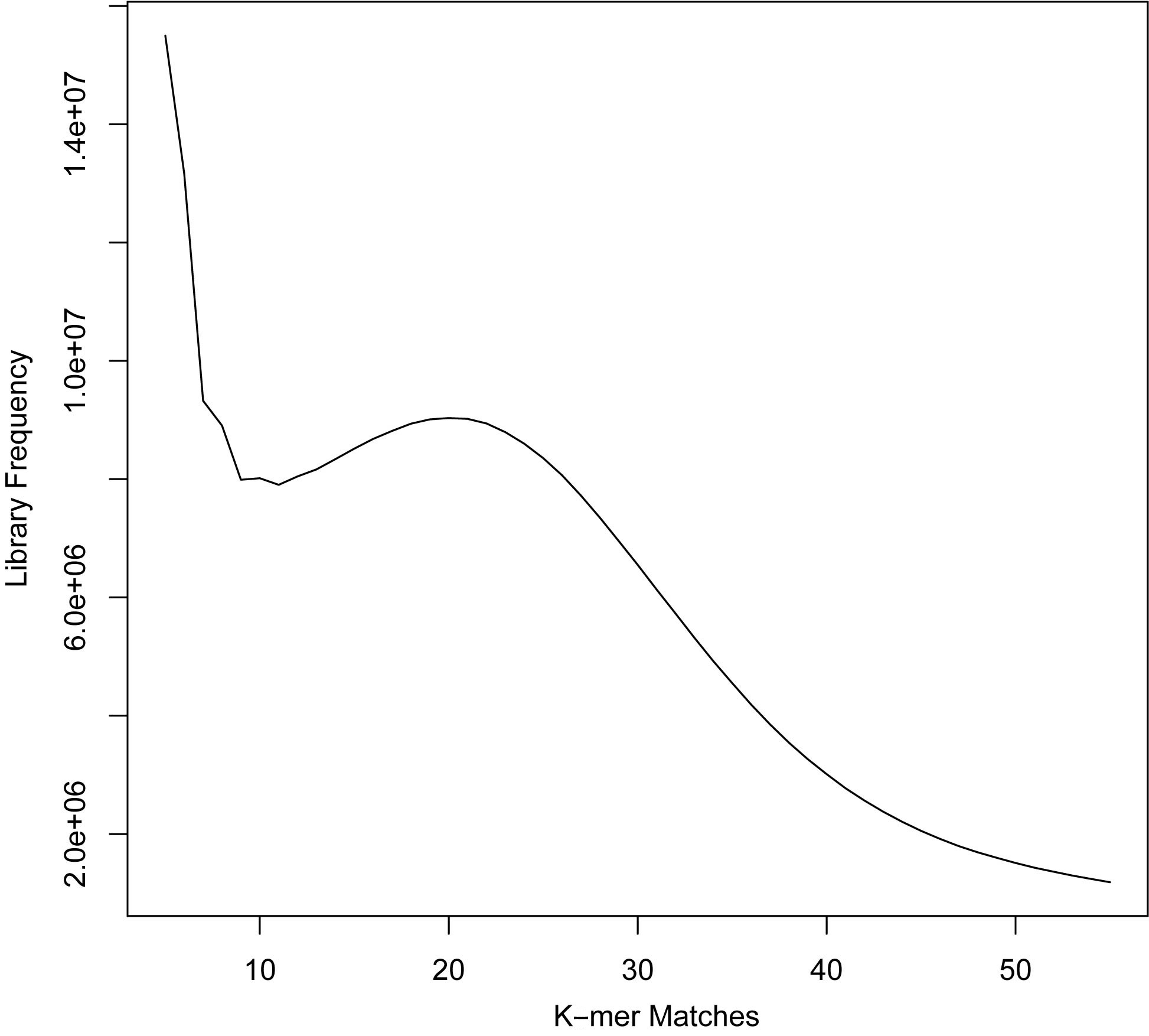

Supplement: FIGURE S1 — 17-k-mer frequency distribution of sequencing reads from M. delavayi. The size of the M. delavayi genome was estimated at 899 Mb. [file Image_1.jpg]

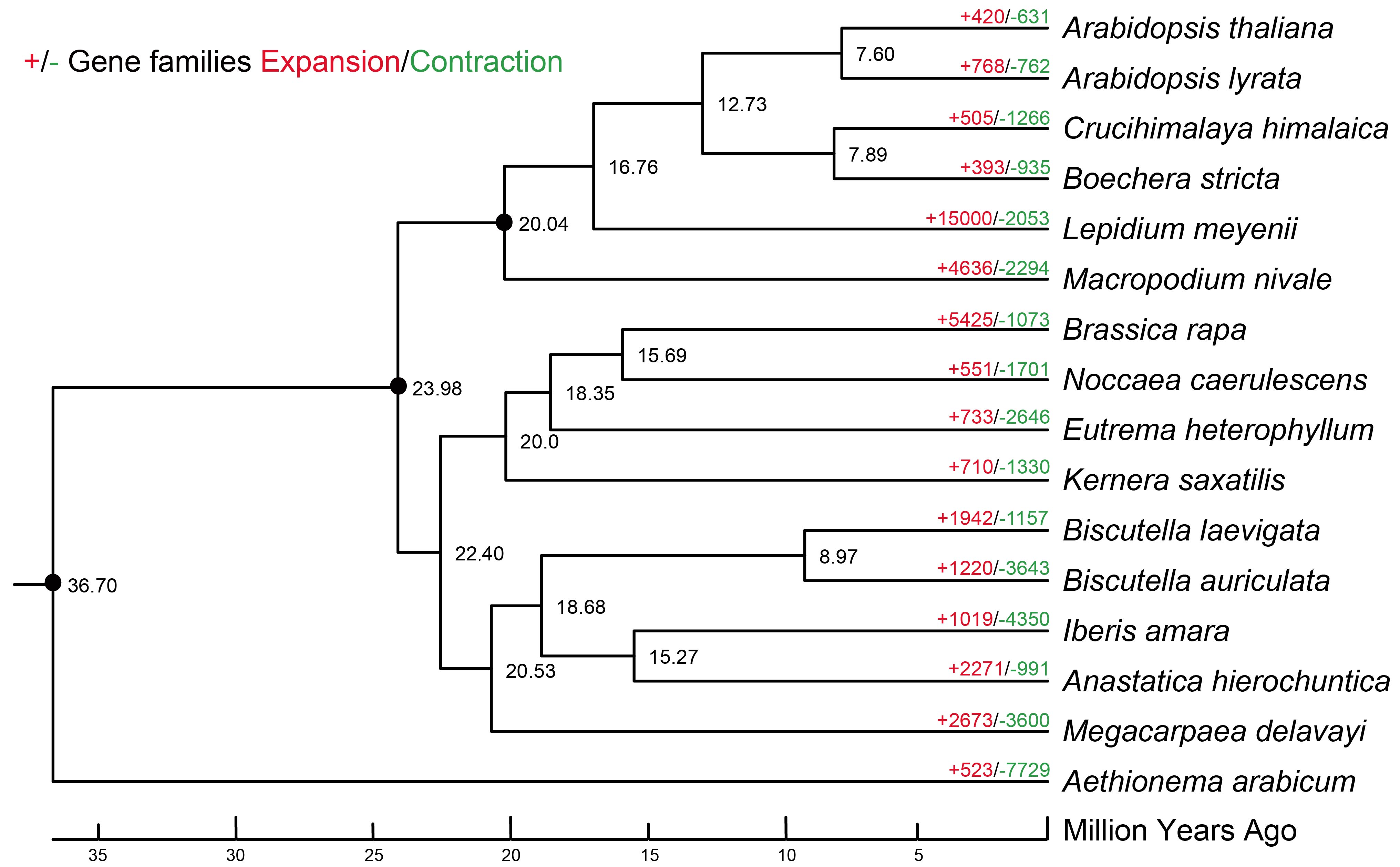

Supplement: FIGURE S2 — Phylogenetic tree with divergence time and expanded/contracted gene family for M. delavayi and other Brassicaceae species. The numbers above the branches are the predicted divergence times. [file Image_2.JPEG]
