# Supplementary material for: The Genome Sequence of Alpine Megacarpaea delavayi Identifies Species-Specific Whole-Genome Duplication
Source: Front Genet. 2020 Aug 3;11:812. doi: 10.3389/fgene.2020.00812 (PMC7416671; doi:10.3389/fgene.2020.00812)
Supplement: TABLE S1 — Sequencing statistics of the M. delavayi genome. [file Data_Sheet_1.zip › Supplementary Tables.DOCX]

Supplementary Table S1: Sequencing statistics of the *M. delavayi* genome

| **Sample** | **Raw Reads** | **Clean Reads** | **Raw Base (G)** | **Clean Base (G)** |
| --- | --- | --- | --- | --- |
| GHCA_230bp | 129,758,627 | 127,532,395 | 38.927 | 38.260 |
| GHCA_500bp | 99,501,537 | 96,838,099 | 29.850 | 29.051 |
| GHCA_800bp | 40,596,427 | 38,971,070 | 12.180 | 11.691 |
| GHCA_2kb | 39,466,007 | 39,451,971 | 11.839 | 11.835 |
| GHCA_5kb | 38,267,004 | 38,254,032 | 11.480 | 11.476 |
| GHCA_10kb | 62,299,622 | 56,662,490 | 18.690 | 16.999 |
| GHCA_20kb | 47,015,121 | 47,001,974 | 14.105 | 14.101 |
| Total | 456,904,345 | 444,712,031 | 137.071 | 133.413 |

Supplementary Table S2: Evaluation *M. delavayi* genome assemby with BUSCO

| **Parameter** | **Number** | **Percentage (%)** |
| --- | --- | --- |
| Complete BUSCOs | 1,360 | 94.5 |
| Complete and single-copy BUSCOs | 1,232 | 85.6 |
| Complete and duplicate BUSCOs | 128 | 8.9 |
| Fragmented BUSCOs | 21 | 1.5 |
| Missing BUSCOs | 59 | 4.0 |
| Total BUSCO groups searched | 1,440 | 100 |

Supplementary Table S3: Intact LTR-RTs in *M. delavayi* and other Brassicaceae species

| **Species** | **Number** | **LTR size(bp)** | **% of genome** |
| --- | --- | --- | --- |
| *Anastatica hierochuntica* | 889 | 218,450,151 | 40.28 % |
| *Arabidopsis thaliana* | 176 | 7,863,937 | 6.60 % |
| *Arabidopsis lyrata* | 1,227 | 32,337,407 | 15.49 % |
| *Brassica rapa* | 213 | 16,546,465 | 5.25 % |
| *Capsella rubella* | 185 | 7,563,566 | 5.64 % |
| *Megacarpaea delavayi* | 814 | 346,444,201 | 39.20% |

Supplementary Table S4: Numbers of annotated protein-coding genes in *M. delavayi* genome

| **Functional Annotations** | **Number of annotated genes** | **Precent of total genes (%)** |
| --- | --- | --- |
| InterProScan | 35,267 | 85.78 |
| Gene Ontology (GO) | 28,966 | 70.53 |
| Swiss-Prot | 26,750 | 65.06 |
| KEGG | 9,868 | 21.81 |
| Total gene | 41,114 | 100 |

Supplementary Table S5: Protein data sets used for comparative genomic analyses

| **Species** | **Version** | **Data source/ GenBank accession number** |
| --- | --- | --- |
| *Arabis alpina* | - | NCBI: JNGA00000000 |
| *Aethionema arabicum* | *-* | NCBI: PRJNA202984 |
| *Anastatica hierochuntica* | - | ENA: PRJEB26555 |
| *Arabidopsis lyrata* | v1.0 | https://phytozome.jgi.doe.gov |
| *Arabidopsis thaliana* | Tair10 | https://phytozome.jgi.doe.gov |
| *Biscutella auriculata* | - | ENA: PRJEB26555 |
| *Biscutella laevigata* | - | ENA: PRJEB26555 |
| *Brassica rapa* | FPsc v1.3 | https://phytozome.jgi.doe.gov |
| *Boechera stricta* | v1.2 | https://phytozome.jgi.doe.gov |
| *Capsella rubella* | v1.1 | https://phytozome.jgi.doe.gov |
| *Crucihimalaya himalaica* | - | https://bioinformatics.psb.ugent.be/gdb/cruhi/ |
| *Eutrema heterophyllum* | - | NCBI: PKMM00000000 |
| *Iberis amara* | - | ENA: PRJEB26555 |
| *Kernera saxatilis* | - | ENA: PRJEB26555 |
| *Lepidium meyenii* | - | http://www.herbal-genome.cn. |
| *Macropodium nivale* | - | ENA: PRJEB26555 |
| *Noccaea caerulescens* | - | ENA: PRJEB26555 |
| *Schrenkiella parvula* | V2.0 | http://thellungiella.org/data |

Supplementary Table S6: Function of significantly expanded genes in *M. delavayi* genome

Supplementary Table S7: GO enrichment of *M. delavayi* specific genes.

Supplementary Table S8: Function of fast-evolving genes in *M. delavayi* genome

Supplementary Table S9: *DRT* genes copy number in *M. delavayi* and *L. meyenii* genomes (Identity >80%)

| ***DRT* genes** | ***Arabidopsis thaliana*** | ***Megacarpaea delavayi*** | ***Lepidium meyenii*** |
| --- | --- | --- | --- |
| *DRT100* | 1 (*AT3G12610*) | 1 | 3 |
| *DRT101* | 1 (*AT5G18070*) | 2 | 3 |
| *DRT102* | 1 (*AT3G04880*) | 0 | 2 |
| *DRT111* | 1 (*AT1G30480*) | 2 | 4 |
| *DRT112* | 1 (*AT1G20340*) | 1 | 3 |
